# Dynamic Changes in DNA Methylation in Ischemic Tolerance

**DOI:** 10.3389/fneur.2015.00102

**Published:** 2015-05-15

**Authors:** Robert Meller, Andrea Pearson, Roger P. Simon

**Affiliations:** ^1^Translational Stroke Program, Neuroscience Institute, Morehouse School of Medicine, Atlanta, GA, USA; ^2^Grady Memorial Hospital, Atlanta, GA, USA

**Keywords:** DNA methylation, ischemia, preconditioning, stroke, ischemic tolerance, ChIP-seq

## Abstract

Epigenetic mediators of gene expression are hypothesized to regulate transcriptomic responses to preconditioning ischemia and ischemic tolerance. Here, we utilized a methyl-DNA enrichment protocol and sequencing (ChIP-seq) to identify patterns of DNA methylation in an established model of ischemic tolerance in neuronal cultures (oxygen and glucose deprivation: OGD). We observed an overall decrease in global DNA methylation at 4 h following preconditioning ischemia (30 min OGD), harmful ischemia (120 min OGD), and in ischemic tolerant neuronal cultures (30 min OGD, 24 h recovery, 120 min OGD). We detected a smaller cohort of hypermethylated regions following ischemic conditions, which were further analyzed revealing differential chromosomal localization of methylation, and a differential concentration of methylation on genomic regions. Together, these data show that the temporal profiles of DNA methylation with respect to chromatin hyper- and hypo-methylation following various ischemic conditions are highly dynamic, and may reveal novel targets for neuroprotection.

## Introduction

Pharmacological agents for neuroprotection have been disappointing in clinical trials. An understanding of the basic mechanisms of endogenous neuroprotection in brain, those induced by preconditioning ischemia and resulting in a tolerant phenotype, may yield novel approaches to reduce brain injury following ischemia (1, 2). We have previously described broad-based transcriptional suppression as operative in ischemic tolerance (3), and have discovered protein effectors of tolerance: upregulation of gene silencing polycomb proteins in tolerant brain (4). Polycomb protein’s mechanism of action is via epigenetic chromatin binding, highlighting a putative biologic mechanism with neuroprotective potential. DNA methylation has been prominently featured as an epigenetic modulator in brain development as well as in brain injury (5, 6).

It has been proposed that a reduction in DNA methylation will confer protection against brain ischemia. Global DNA methylation increases in the brain following harmful focal ischemia, as measured by [^3^H]-methyl incorporation into DNA (7), but does not change the expression of DNMT1 or 3 (DNA methyltransferase, DNMT). However, the chromatin sequences that are methylated by ischemia were not identified by Endres (7). Blocking the enzyme responsible for DNA methylation, DNMT, using 5′ azacytidine inhibits ischemic cell death (7). Heterozygous, but not homozygous knockouts of DNA methyltransferase1 also show a reduction in brain infarction following modeled ischemia (8). Therefore, reducing DNA methylation may protect the brain from subsequent harmful ischemia.

An increase in DNA methylation following ischemia would be consistent with a recent study which showed that MECP2 (methyl CpG binding protein 2), a transcription repressor, is increased following brief ischemia (9). In addition, the DNA methylating agent methylazoxymethanol (MAM) blocks ischemic tolerance induced neuroprotection (10). In that study, while the effect was attributed to a blockade in progenitor cell proliferation, the ability of MAM to hypermethylate DNA such that preconditioning-induced changes in DNA methylation are blocked, cannot be ruled out. While it is unclear which genes are methylated in response to ischemia, ischemia can affect DNA methyltransferase activity in brain, and a reduction in DNA methylation may play a role in mediating the protective effects of ischemic tolerance.

The role of DNA methylation was recently studied in an analogous model of ischemic tolerance that of tolerance to seizure-induced brain injury (11). Following preconditioning seizures, brain injury following status epilepticus induced by an intra-amygdaloidal injection of kainic acid is reduced, specifically in the CA3 subfield of the hippocampus (12–14). Similar to ischemic tolerance, a reduction in gene expression has been reported in seizure tolerant brain, suggesting gene repression mediates tolerance to both seizure and ischemic brain injury (13). In the seizure tolerance study, DNA methylation patterns were profiled at 8 h post seizure, and revealed a dynamic pattern whereby both an increase and decrease in methylation were observed (11).

Thus, the role of DNA methylation in ischemic tolerance is not clear. Here, we offer an in-depth analysis of DNA methylation in a cell culture model of ischemic preconditioning-induced ischemic tolerance (15).

## Materials and Methods

### Cell culture and ischemic tolerance modeling

All animal use was approved by the Morehouse School of Medicine Institutional Animal Care and Use Committee. Primary neuronal cultures were prepared for mixed sex litters of rats (1 day post birth). Animals were euthanized with isoflurane, and cortices dissected in an ice cold buffer (dissociation media). Tissue was incubated with papain (37°C: 10 min) and triturated. Cells were seeded out a 5 million cells/6 cm dish. Cells were grown in a mixture of B27 (3.5%) and Neurocult (1.5%) supplemented NeuroBasal A media. Cells were used at 14 days *in vitro* (DIV).

An *in vitro* equivalent of ischemic tolerance is used, whereby primary neuronal cultures are subjected to oxygen and glucose deprivation (OGD) modeled ischemia (16–18). Cells are washed with phosphate buffered saline, supplemented with Mg Cl_2_ and Ca Cl_2_, and then incubated for 30 or 120 min in a Thermofisher anaerobic chamber. Cells were recovered in neurobasal A media. For modeling ischemic tolerance, we precondition cells with 30 min OGD, and injurious ischemia (120 min) is applied 24 h later. Cells were harvested 4 h following the final ischemic challenge.

### DNA preparation and methylation enrichment

Methylated DNA was enriched from cell lysates (*n* = 2/condition) using the Methylminer assay (Active Motif). DNA was extracted from frozen cell pellets using the Genelute DNA Kit (Sigma). DNA was quantified and 2.0 μg was sonicated in 200 μl Tris EDTA buffer (1 × 30% 10 s, 2 × 20% 10 s, 4°C). Sheared DNA (2000 ng) was used as starting material for the pull down reaction according to manufacturer’s protocol. Following pull down, the DNA was size selected on a 1% agarose gel [200–500 base pair (bp) fragments] and extracted using a PCR (polymerase chain reaction) clean-up kit (Qiagen).

### DNA library preparation

**Figure 1:**
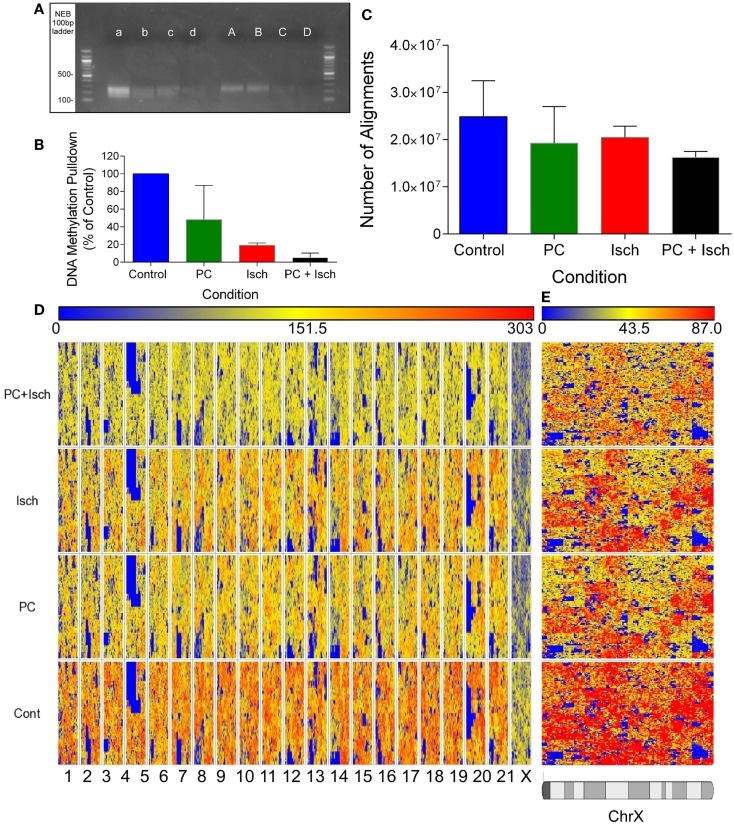
**Global decreases in DNA methylation revealed by DNA sequencing**. **(A)** Agarose Gel showing approximate size of prepared libraries following enrichment of methyl-DNA using the methylminer assay. Libraries were amplified using PCR prior to running the gel. A–d refer to two independent experiments, A, a Cont, B, b-PC, C, c-Isch, D, d-PC + Isch. **(B)** Optical quantification of bands in **(A)** using Kodak Biostation 4000MM. **(C)** Quantification of number of reads aligned to the Rn5 reference genome using Lifescope software. Data are *n* = 2, mean ± SD. **(D)** Partek generated Hilbert graph showing methylation across the rat genome. Chromosome location is represented on the bottom of the figure and conditions are denoted on the left. Higher methylation is represented by warm colors (red) and hypomethylation by blue. **(E)** Expanded view of Chromosome X Hilbert Graph, showing representative decrease in methylation across all treatments compared to control.

Samples were end-polished and ligated to barcode adapters using T4 Kinase. Libraries were amplified using Platinum taq for 12 cycles of PCR, size selected on a 2% agarose gel, and then subjected to an additional four cycles of PCR. Libraries were visualized on an agarose gel (see Figure 1A), excised, and cleaned up to remove primers (Qiagen). Samples were then subjected to qPCR to quantify the library against a known standard. Emulsion PCR was used to clone the libraries onto sequencing beads. The reaction was seeded at 1.0 pmol of equimolar library (all eight samples at the same concentration). A WFA run determined the final concentration of beads for deposition on the sequencing slide to obtain approximately 700 million beads deposited on the slide. Libraries were sequenced on an Applied Biosystems SOLiD 4 DNA sequencer using a F35 single end read. Data were stored as csfasta files, and uploaded to a penguin cluster running Lifescope^TM^ for alignment to the RN5 genome (rat), using the RefSeq annotation guide (October 2014).

### Data analysis

Data were transferred to a Dell Dimension Desktop computer running Partek Genomic Studio v 6.6 for analysis. Data were analyzed using the Partek ChIP and the NGS Methylation workflows. Output bam files were combined according to experimental conditions for all analysis. Data were subjected to cross strand analysis to identify the fragment window size for genomic analysis (Figure 1C). Data were aligned to the rn5 genome, using the control sample as the reference, and a window bin size of 200 bp. Only fragments that show enrichment with a *p* < 0.001 false detection rate (FDR) were considered significant and identified in a “peaks file.” These data were used for subsequent analysis (available upon request).

## Results

### Ischemic stimuli result in a reduction in global DNA methylation

Methylation profiles of ischemia treated neuronal cell cultures were identified using methylated chromatin enrichment followed by DNA sequencing (ChIP-seq). We analyzed four treatment groups: sham-treated cells (Control: Cont), cells subjected to 30 min OGD (Preconditioning: PC) and recovered for 4 h, cell subjected to 120 min ischemia (Injurious Ischemia: Isch), and neurons subjected to 30 min OGD and recovered 24 h prior to harmful ischemia (Tolerance: PC + Isch). These conditions have been established in multiple studies as showing ischemic tolerance (3, 16, 18). We focused on a time point of 4 h following the last ischemic treatment to identify dynamic changes in methylation that may be responsible for subsequent gene expression changes.

Quantitative analysis of assembled libraries on an agarose gel suggests a global decrease in DNA methylation, detected using the Methylminer assay. Interestingly, methylation was reduced following all ischemia treatment protocols (Figures 1A,B). Libraries were assembled and DNA concentrations were normalized prior to sequencing. The sequenced data were aligned to the rn5 reference genome; there were similar numbers of alignments in each experimental group (mean of 20.2 million reads ± 1.8 million reads; Figure 1C). The equivalent number of aligned reads is due to the concentration normalization step in the sequencing protocol. Therefore, all subsequent analysis reveals differences of methylation enrichment patterns rather than absolute DNA methylation levels.

In order to compare datasets, we combined the reads from each experimental condition (Cont, PC, Isch, PC + Isch), resulting in 30–40 million aligned reads per condition. Global methylation status was visualized with a Hilbert graph (19). Intensity of methylation is represented by red, and weaker intensity is represented as blue. Similar to previous published studies, the visualization of DNA methylation patterns showed a diffuse pattern of reads across the genome (Figure 1D). However, it is of note that the intensity of reads is lower globally across the genomes of the PC, Ischemia, and PC + Ischemia treated cells, consistent with the library analysis (Figure 1A). Of note, we also observed a lower global representation of methylation on the X chromosome (Figure 1E).

**Figure 2:**
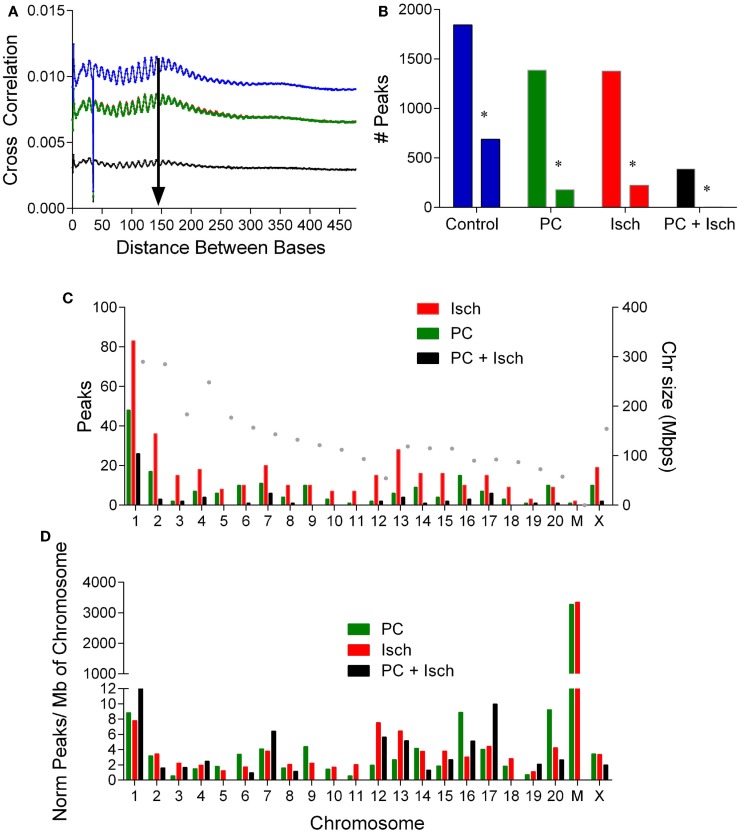
**Analysis of DNA methylation peaks reveals differential chromosomal methylation**. **(A)** Cross strand analysis to determine library size for peak analysis. The PC and ischemia curves overlap (Cont: blue, PC: green, Isch: red, and PC + Isch: black). **(B)** Quantification of number of peaks in each treatment condition, and the number of unique peaks in each condition denoted by an asterisk. Data from the two sets were combined. **(C)**. Distribution of raw peak occurrence by chromosome for PC, Ischemia, and PC + Isch treated cells. The size of the chromosome is plotted on the right axis. **(D)**. Normalization of chromosome location data, by number of peaks and per Mbp of chromosome to reveal selective enrichment of chromosome patterns of DNA methylation. Note high relative methylation of mitochondria (discontinuous axis).

A peak analysis was performed on the DNA-seq data to identify clusters of methylation and relative differences in methylation compared to control. The window for the fragment size for analysis was determined by performing a cross strand analysis (20) (Figure 2A). Samples showed an approximate peak of around 150 bp (arrow Figure 2A). Reads were analyzed using a 150 bp fragment window, and using the control samples as a reference (to compare DNA methylation to control). The genome was divided into 100 bp windows, which could be combined. Data were fit to a negative binominal model with a peak cutoff false detection rate (FDR) of *p* < 0.001. From this analysis, 5035 peaks were identified in each of the four conditions. When we plot the number of methylation peaks (Figure 2B), we observed that the number of peaks decreases in the PC, Ischemia, and PC + Ischemia treated cells. The number of peaks unique to each condition are also plotted, and these have a similar distribution (peaks unique to each condition are marked with an asterisk; Figure 2C). Interestingly, although global methylation is reduced in ischemia-treated cells compared to PC-treated cells (Figure 1B), the number of hyper methylated regions is the same (Figure 2B). Our interpretation of this result is that global levels of DNA methylation are reduced 4 h following harmful ischemia, but individual genes may show a strong increase in methylation, which we further investigated (below).

### Dynamic changes in methylation following ischemia show chromosomal and functional bias

In order to identify genes whose methylation increased following ischemia treatments, we subjected the data to a one-tailed negative binominal test and selected a cut off FDR value of 0.05. Compared to the control sample, 618 regions show significant enrichment, which we interpreted as increased methylation. There were more enriched regions in the ischemia group (345) compared to those treated with preconditioning only (187), or in tolerant samples (65) (Figure 2D).

We separated the differentially methylated peaks by treatment, and identified which genomic feature they were close to and which chromosome they align with. Analysis of the enriched methylation peak distribution revealed an increase in chromosome 2, 12, and 13 methylation in the ischemia-treated cells (Figure 2C). In order to assess relative chromosomal distribution, we normalized the number of peaks to the total number of peaks/condition, and then scaled to the size of the chromosome (Figure 2D). Global methylation was reduced following PC + Ischemia/tolerance (Figure 1B); it is of note that chromosome 1, 7, and 17 showed relative enhancements in hypermethylation compared to control. Following PC, methylation of chromosome 6, 9, 16, and 20 showed enhanced hypermethylation compared to control. There was no chromosome selective enrichment of hypermethylated regions in ischemia-treated cells; rather, hypermethylated chromosomes were common preconditioned cells (chromosome 2 and chrX), or preconditioned cells subjected to harmful ischemia (chromosome 3, 12, and 13) (Figure 2D). This suggests that different chromosomes show predominant methylation in response to various ischemic treatments, which may result in differential activation of gene expression/repression in response to these stimuli.

**Figure 3:**
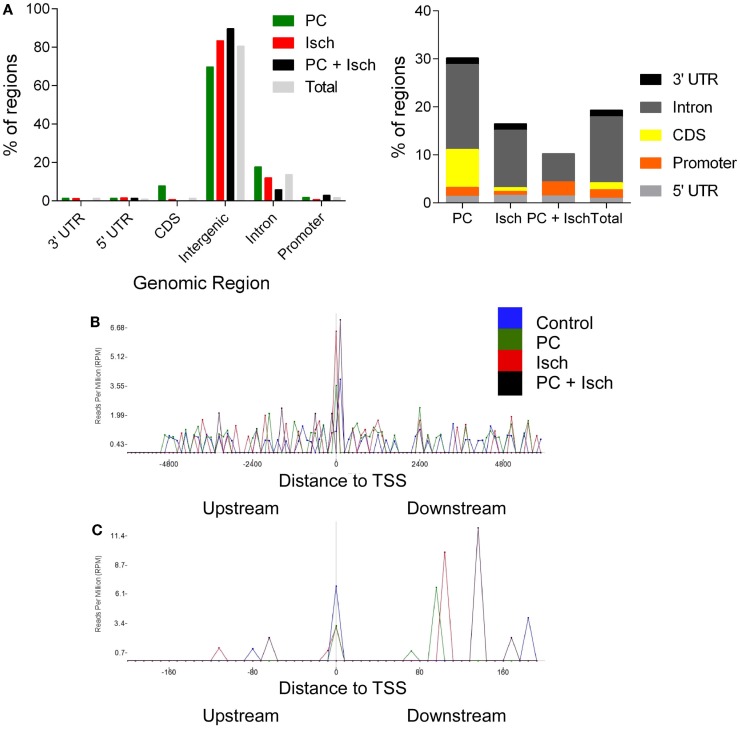
**Genomic location of DNA methylation following ischemia treatments**. **(A)** Genomic locations of peaks as identified with RefSeq annotation guide. Most DNA methylation peaks are deemed intergenic, or between genes. Total represents a combination of all of the peaks and then analyzed for genomic location. Enlargement of non-intergenic associating reads, grouped by condition [N.B. re-draw of data in **(A)**]. **(B)** Analysis of reads relative to the transcription start site (+6000 to −6000 bases). Note the uniform distribution except at the TSS. The data are analyzed by determining the frequency of reads in 100 bins across the range of the *x* axis. **(C)** Enlargement of the −22 to +200 base region around the transcription start site.

The lists of significantly regulated genes were then analyzed, according to which genomic feature they correspond with using the RefSeq Transcripts Annotation guide (24 October, 2014) (Figure 3A). Promoters were defined as 5000-0 bases downstream of the transcription start site. Data from multiple introns and exons (CDS) were combined into one category. The most notable feature was the very high representation of intergenic regions in this data set; yet, only 1% of the genome encodes genes. It is not clear whether these intergenic associated regions of methylation are associated with non-transcribed DNA or novel transcripts of RNA, which are as yet unannotated (or not in the refSeq guide). Ischemia-treated and tolerant cells appear to have similar concordance with a control (total) distribution of methylation across genomic features. In contrast, following preconditioning ischemia, there was a relative increase in PC-mediated exon and intron-associated methylation, and a decrease in intergenic methylation (Figures 3A,B).

Finally, we investigated the location of reads relative to the transcription start site (TSS). This analysis is independent of the gene name; so, it only considers the relative location of peaks to the closest TSS. All samples had a similar profile in that we saw a relatively even distribution before and after the TSS (Figure 3B). A notable exception is at the actual TSS where the depth of reads is higher. When we expand this region (Figure 3C), we observed that control, PC, and ischemia treated samples showed a clustering around the TSS; in contrast, the largest peak from the tolerant cells was approximately 150 bp downstream of the TSS. Whether this was due to the loss of global methylation in these samples or a more selective methylation mark close to the TSS is not yet clear. Similarly, samples from PC and Ischemia treated cells also show a peak, but closer to 100 bp downstream of the TSS. The significance of these marks on gene expression awaits further investigation.

### Pathway analysis of methylated regions

For each treatment group, we created lists of genes closest to the methylation peaks, and genes unique to each treatment group. We performed a pathway analysis on the gene lists associated with methylated regions. Fisher’s exact test was performed, and pathways were restricted to have at least two genes present. From this analysis, we first looked at enrichment scores and enrichment *p*-values, with a cut off of *p* < 0.05. (Table 1A depicts the pathways associated with the methylated genes in control, and 1B unique to control). In control samples, 13/19 of the pathways were associated with immune and inflammatory responses, or were identified as having genes associated with the immune system. Unique to control, we observe 19 pathways showing enrichment, again showing a high number of immune system associated pathways (Table 1B).

**Table 1:** **(A) List of pathways identified from closest gene to methylation peaks in control MeDNA sample; (B) list of pathways identified from closest gene to methylation peaks unique to control MeDNA sample**.

Pathway name	Database	Enrichment score	Enrichment *p*-value	% genes in pathway that are present	Sample ID vs. cont score	# genes in list, in pathway
**(A)**						
Allograft rejection	kegg	17.17	3.4889E-08	11.48	0.62	7
Autoimmune thyroid disease	kegg	16.61	6.1039E-08	10.61	0.62	7
Viral myocarditis	kegg	15.35	2.156E-07	8.86	0.62	7
Antigen processing and presentation	kegg	14.60	4.5532E-07	7.95	0.62	7
Graft-versus-host disease	kegg	14.09	7.6324E-07	10.17	0.62	6
Cell adhesion molecules (CAMs)	kegg	13.26	1.7457E-06	5.16	0.62	8
Type I diabetes mellitus	kegg	13.23	1.7874E-06	8.82	0.62	6
Phagosome	kegg	10.21	3.6978E-05	4.12	0.61	7
Epstein–Barr virus infection	kegg	8.86	0.00014	3.33	0.62	7
Endocytosis	kegg	8.69	0.00017	3.24	0.62	7
Herpes simplex infection	kegg	7.62	0.00049	3.28	0.62	6
HTLV-I infection	kegg	7.39	0.00062	2.62	0.62	7
Viral carcinogenesis	kegg	7.18	0.00076	3.02	0.62	6
Natural killer cell mediated cytotoxicity	kegg	4.37	0.01264	3.45	0.53	3
Linoleic acid metabolism	kegg	4.22	0.01464	6.06	0.34	2
Lysine degradation	kegg	3.77	0.02312	4.76	0.47	2
Serotonergic synapse	kegg	3.66	0.02585	2.63	0.33	3
Hippo signaling pathway	kegg	3.13	0.04357	2.14	11.40	3
Synthesis and degradation of ketone bodies	kegg	3.00	0.04965	11.11	0.57	1
**(B)**						
Allograft rejection	kegg	20.47	1.29E-09	11.48	0.26	7
Autoimmune thyroid disease	kegg	19.90	2.27E-09	10.61	0.26	7
Viral myocarditis	kegg	18.62	8.22E-09	8.86	0.26	7
Antigen processing and presentation	kegg	17.85	1.76E-08	7.95	0.26	7
Graft-versus-host disease	kegg	16.84	4.84E-08	10.17	0.26	6
Type I diabetes mellitus	kegg	15.98	1.15E-07	8.82	0.26	6
Cell adhesion molecules (CAMs)	kegg	13.94	8.85E-07	4.52	0.26	7
Epstein–Barr virus infection	kegg	11.90	6.77E-06	3.33	0.26	7
Endocytosis	kegg	11.72	8.16E-06	3.24	0.26	7
Phagosome	kegg	10.59	2.53E-05	3.53	0.26	6
HTLV-I infection	kegg	10.33	3.26E-05	2.62	0.26	7
Herpes simplex infection	kegg	10.17	3.84E-05	3.28	0.26	6
Viral carcinogenesis	kegg	9.70	6.15E-05	3.02	0.26	6
Linoleic acid metabolism	kegg	5.06	0.0063	6.06	0.27	2
Arachidonic acid metabolism	kegg	3.55	0.0288	2.74	0.27	2
Retinol metabolism	kegg	3.50	0.0302	2.67	0.27	2
Steroid hormone biosynthesis	kegg	3.47	0.0310	2.63	0.27	2
Chemical carcinogenesis	kegg	3.36	0.0348	2.47	0.27	2
Natural killer cell mediated cytotoxicity	kegg	3.23	0.0397	2.30	0.27	2

Following preconditioning ischemia, 17 pathways are identified, 10 of which are immune system, but 2 are associated with serotonin (Table 2). In the unique PC gene list, two-third of the pathways identified with synaptic pathways rather than the immune system. Following harmful ischemia (Tables 3A,B), 15 pathways showed enrichment, of which 13 are immune function associated. When we look at pathways unique to ischemia, three pathways associated with the synapse and receptor ligand interactions are identified. In ischemic tolerance, we do not observe any significant pathways identified, but one pathway, insulin secretion, was identified in the unique tolerance sample (Table 4). The low number of pathways identified in the tolerant sample is due to the low number of methylation associated peaks identified in these samples.

**Table 2:** **(A) List of pathways identified from closest gene to methylation peaks in PC MeDNA sample; (B) list of pathways identified from closest gene to methylation peaks unique to PC MeDNA sample**.

Pathway name	Database	Enrichment score	Enrichment *p*-value	% genes in pathway that are present	Sample ID vs. cont score	# genes in list, in pathway
**(A)**						
Serotonergic synapse	kegg	8.08	0.00031	4.39	1.13	5
Graft-versus-host disease	kegg	5.60	0.003684	5.08	0.74	3
Allograft rejection	kegg	5.51	0.004049	4.92	0.74	3
Autoimmune thyroid disease	kegg	5.29	0.005055	4.55	0.74	3
Type I diabetes mellitus	kegg	5.20	0.005496	4.41	0.74	3
Viral myocarditis	kegg	4.79	0.008333	3.80	0.74	3
Antigen processing and presentation	kegg	4.49	0.011193	3.41	0.74	3
Phagosome	kegg	4.41	0.012174	2.35	0.71	4
HTLV-I infection	kegg	4.36	0.01282	1.87	0.87	5
Linoleic acid metabolism	kegg	4.33	0.013157	6.06	1.17	2
Retrograde endocannabinoid signaling	kegg	4.18	0.015373	3.03	1.20	3
Herpes simplex infection	kegg	4.16	0.015608	2.19	0.80	4
Tryptophan metabolism	kegg	3.83	0.021766	4.65	0.98	2
Endocytosis	kegg	3.62	0.026903	1.85	0.80	4
Ovarian steroidogenesis	kegg	3.55	0.028851	4.00	0.81	2
Synthesis and degradation of ketone bodies	kegg	3.06	0.04702	11.11	0.57	1
Cell adhesion molecules (CAMs)	kegg	3.02	0.048765	1.94	0.74	3
**(B)**						
Retrograde endocannabinoid signaling	kegg	3.633	0.026	2.020	2.506	2
HTLV-I infection	kegg	3.518	0.030	1.124	2.922	3
Serotonergic synapse	kegg	3.372	0.034	1.754	2.126	2
Dorso–ventral axis formation	kegg	2.936	0.053	4.762	5.644	1

**Table 3:** **(A) List of pathways identified from closest gene to methylation peaks in ischemia MeDNA sample; (B) List of pathways identified from closest gene to methylation peaks unique to ischemia MeDNA sample**.

Pathway name	Database	Enrichment score	Enrichment *p*-value	% genes in pathway that are present	Sample ID vs. cont score	# genes in list, in pathway
**(A)**						
Cell adhesion molecules (CAMs)	kegg	6.42	0.0016	3.226	0.905	5
Serotonergic synapse	kegg	5.61	0.0037	3.509	2.831	4
Graft-versus-host disease	kegg	5.44	0.0043	5.085	0.785	3
Allograft rejection	kegg	5.35	0.0047	4.918	0.785	3
Autoimmune thyroid disease	kegg	5.13	0.0059	4.545	0.785	3
Type I diabetes mellitus	kegg	5.05	0.0064	4.412	0.785	3
Viral myocarditis	kegg	4.63	0.0097	3.797	0.785	3
Natural killer cell mediated cytotoxicity	kegg	4.37	0.0126	3.448	1.242	3
Antigen processing and presentation	kegg	4.34	0.0130	3.409	0.785	3
Linoleic acid metabolism	kegg	4.22	0.0146	6.061	1.969	2
Phagosome	kegg	4.22	0.0148	2.353	0.767	4
Neuroactive ligand-receptor interaction	kegg	3.94	0.0194	1.786	6.661	5
Glutamatergic synapse	kegg	3.80	0.0225	2.778	11.711	3
Endocytosis	kegg	3.43	0.0323	1.852	0.816	4
Ribosome	kegg	3.36	0.0348	2.344	3.650	3
**(B)**						
Neuroactive ligand-receptor interaction	kegg	6.94	0.000969	1.79	10.74	5
Glutamatergic synapse	kegg	5.66	0.003468	2.78	11.71	3
GABAergic synapse	kegg	3.68	0.025125	2.33	9.56	2

**Table 4:** **List of Pathways identified from closest gene to methylation peaks unique to tolerance MeDNA sample**.

Pathway Name	Database	Enrichment score	Enrichment *p*-value	% genes in pathway that are present	Sample ID vs. cont score	# genes in list, in pathway
Insulin secretion	kegg	4.35823	0.012801	1.19048	18.4401	1

## Discussion

Methylation of DNA is an important mechanism by which epigenetic modulation of gene expression occurs. Here, we show that both protection inducing ischemia (preconditioning) and harmful ischemia reduce global DNA methylation. The decrease in global methylation was more pronounced in ischemic tolerant cells. While global methylation was reduced, we observe an increase in methylation in chromosome 2, 12, and 13 in ischemia-treated cells and chromosome 1, 7, and 17 in tolerant cells, suggesting selective enhancement/targeting of methylation at these chromosomes. While the distribution of methylation across ischemic and tolerant genomic regions was similar in incidence, in the preconditioned cells an exon associated methylation induction and attenuation in intergenic methylation were noted. A clustering of differential methylation at transcriptional start sites was observed in all cell treatment groups.

Similar global changes in methylation are reported in brain following injurious status epilepticus when compared to changes seen after induction of seizure tolerance (11). The hypothesis in both our ischemia studies and those of seizure was that the activation of neuroprotective mechanisms via previously described genomic reprograming and transcriptional suppression might occur via epigenetic processes, specifically by DNA methylation. However, in both our study of ischemia and the seizure study, a profound global hypomethylation event was observed in response to cell stress (ischemia or seizures). While this contrasts with studies showing increased methylation following harmful ischemia (7), it should be noted that the present tolerance and seizure studies used shorter recovery times following the stress 4 h (present study) and 8 h (11). It should also be noted that 4 h following preconditioning ischemia and preconditioning seizures, we have noted larger changes in gene expression compared to 24 h post preconditioning [(21) and Meller, unpublished].

Global demethylation was more pronounced in the ischemic tolerant group (cells subjected to preconditioning and harmful ischemia) compared to the ischemia or PC only-treated cells. It is not clear whether the reduction in methylation is due to the repeated exposure of the tissue to ischemic stress, resulting in an additive effect, or whether it is unique to the tolerance phenomenon. Hypermethylation was also reduced in this group compared to the other ischemia treatments. Since fewer “enriched” hypermethylation peaks were identified in the tolerance group, our mapping software only found one regulated pathway (insulin secretion). A reduction in hypermethylation in ischemic tolerance was, perhaps, unexpected given the established observations of gene silencing in ischemic tolerance. Indeed, on first appearance our data appear to contradict the hypothesized dogma that ischemic tolerance is associated with gene silencing (3, 4). However, care should be taken in interpreting these data sets together in this way, given that the current ischemic tolerance study identified chromatin methylation status 4 h following the final harmful insult, whereas gene expression and protein levels were reported 24 h following the final insult (3, 4). These discrepancies clearly support the need for further detailing the temporal profiles of both gene expression and chromatin methylation in response to such stimuli.

Most of the differential methylation is intergenic, which could associate with either non-transcribed DNA regions, or currently unannotated RNA species in the genome. RNA sequencing based transcriptome analysis shows approximately 30% of RNA is from such unannotated regions (22). It is interesting that there is a reduction in methylation in these regions of PC treated cells. When we remove the intergenic reads (Figure 3B), we observed that following PC there is a relative increase in methylation in coding regions compared to harmful ischemia in which methylation was focused more in intergenic regions. Whether this suggests changes in structural organization of chromatin or non-coding RNAs remains to be determined. In tolerant cells, we observed a reduction in coding region associated with methylated peaks. This may be due to the lower number of reads in this sample set, or a genuine decrease in exon associated methylation. Additionally, methylation of intronic regions was increased following PC. This may be due to non-coding RNAs associated with intronic regions, or may represent regulation of alternative splicing of gene transcripts, which may occur following preconditioning (Meller, unpublished observation) or ischemia (23).

Following ischemia treatments, we observed a chromosomal difference in patterns of enhanced methylation compared to control cells. This suggests that chromosomal specific patterns of chromatin methylation may be regulated following various ischemic stimuli. How specific chromosomes are targeted for enhanced methylation is not clear, but studies of methylation following seizures support differential chromosomal location of methylation events following stress (11). Our analysis focused on specific increases in methylation clusters, because we observed global decreases in methylation events. Chromosomal specific gene expression effects of excitotoxicity have been reported previously (24). Chromosomal bias may be associated with movement of chromosomes into active nuclear territories (25). For example, chromosome X movement has been described following epilepsy (26). Changes in nuclear structure and epigenetic marks have been reported in response to synaptic and excitotoxic signaling (27).

Our study may upon first analysis appears to contradict the study of Endres; however, it should be noted that very different methodologies were utilized in these studies. In the Endres study, the incorporation of radiolabeled methyl groups into genomic DNA was measured. As such increased methylation may be due to dynamic DNA methylation or DNA methylation following DNA damage and repair, which has been reported following ischemia (28, 29). We also observed a global decrease in methylation following harmful ischemia, and DNA damage would be expected following harmful ischemia, hence re-methylation may occur. DNA methylation blockers are neuroprotective against ischemia (7). The agents were administered 10 min prior to harmful ischemia. However, the temporal profile of protection afforded by such blockade is not clear. In our study, we observed a reduction in global DNA methylation in tolerant neurons, suggesting that preventing the increased specific methylation observed following harmful ischemia could be protective.

In contrast, our study correlates with the observations of Miller-Delaney that DNA hypomethylation is associated with neuroprotection. In the current study, a methyl DNA binding protein related to the methyl binding domain of MeCP2 was used to enrich chromatin, in contrast to an anti-Me-cytosine antibody enrichment procedure (11). Increased MeCP2 has been reported in brain 24 h following preconditioning ischemia due to the loss in the levels of miR-132 (9). Thus, some of the differences in individual gene/chromatin region identification may be due to methods of enrichment. We also utilized DNA sequencing of the whole rat genome vs. mouse promoter arrays (11), which may also reveal differences between studies. Furthermore, sequencing libraries were normalized to approximate equimolar concentrations, prior to seeding the cloning PCR. The effect of this is to ensure even representation of the libraries in the sequencing reactions. However, as a result, some of the global decreases in methylation become less apparent. This was in part controlled for by comparing the methylation to the control sample, thereby revealing the dynamic changes in methylation. Clearly, more analysis of such studies will reveal additional information, especially when more advanced analysis methodologies and tools become available to incorporate such aspects of sequencing.

In summary, we performed a DNA methylation study using next generation sequencing which revealed dynamic changes in DNA methylation following preconditioning ischemia and in ischemic tolerance. Our study showed that 4 h following preconditioning ischemia, harmful ischemia, and in tolerant cells, DNA hypomethylation predominates as the response. However, selective and significant increases in hypermethylation events can also be observed, especially in response to harmful ischemia. The consequence of these events upon transcription awaits definition, but this study suggests that the temporal profile of epigenetic regulation of gene expression events needs further study to improve our understanding of this critical transcription control mechanism, and the identification of novel targets for neuroprotection from stroke induced brain injury.

## Conflict of Interest Statement

The authors declare that the research was conducted in the absence of any commercial or financial relationships that could be construed as a potential conflict of interest.
